# Impact of dysfunctional maternal personality traits on risk of offspring depression, anxiety and self-harm at age 18 years: a population-based longitudinal study

**DOI:** 10.1017/S0033291717001246

**Published:** 2017-06-06

**Authors:** R. M. Pearson, A. Campbell, L. M. Howard, M. H. Bornstein, H. O'Mahen, B. Mars, P. Moran

**Affiliations:** 1 Centre for Academic Mental Health, School of Social and Community Based Medicine, University of Bristol, Bristol, UK; 2 Women's Mental Health, Kings College London, London, UK; 3 Eunice Kennedy Shriver National Institute of Child Health and Human Development, Bethesda, MD, USA; 4 Mood Disorders Centre, University of Exeter, Exeter, UK

**Keywords:** ALSPAC, longitudinal, maternal, mental Health, personality

## Abstract

**Background:**

The impact of underlying parental psychological vulnerability on the future mental health of offspring is not fully understood. Using a prospective cohort design, we investigated the association between dysfunctional parental personality traits and risks of offspring self-harm, depression and anxiety.

**Methods:**

The association between dysfunctional parental personality traits (monotony avoidance, impulsivity, anger, suspicion, and detachment), measured in both mothers and fathers when offspring were age 9 years, and risk of offspring depression, anxiety and self-harm at age 18 years, was investigated in a population-based cohort (ALSPAC) from over 8000 parents and children.

**Results:**

Higher levels of dysfunctional maternal, but not paternal, personality traits were associated with an increased risk of self-harm, depression, and anxiety in offspring. Maternal associations were best explained by the accumulation of dysfunctional traits. Associations were strongest for offspring depression: Offspring of mothers with three or more dysfunctional personality traits were 2.27 (1.45–3.54, *p* < 0.001) times as likely to be depressed, compared with offspring of mothers with no dysfunctional personality traits, independently of maternal depression and other variables.

**Conclusions:**

The accumulation of dysfunctional maternal personality traits is associated with the risk of self-harm, depression, anxiety in offspring independently of maternal depression and other confounding variables. The absence of associations for equivalent paternal traits makes a genetic explanation for the findings unlikely. Further research is required to elucidate the underlying mechanism. Mothers with high levels of dysfunctional personality traits may benefit from additional support to reduce the risk of adverse psychological outcomes occurring in their offspring.

## Introduction

Common mental disorders, notably depression, are among the leading causes of global disease burden (Whiteford *et al.* 2015). Understanding childhood risk factors for these disorders is essential to develop effective prevention and intervention strategies. A number of maternal characteristics are acknowledged risk factors for offspring mental health disorders, including young maternal age, low maternal education and income, and maternal mental health problems (Stein *et al.* 2014). Risk associated with such maternal factors appears to operate, at least in part, through the link between these characteristics and parenting style. Problematic parenting behaviours are, in turn, associated with offspring risk of developing mental health disorders (Kazdin, 1997; Collins *et al.* 2000). To date, some key maternal characteristics remain relatively overlooked. One prominent candidate is maternal personality. The American Psychological Association defines personality as ‘individual differences in characteristic patterns of thinking, feeling and behaving’. Personality determines behaviours towards others, and so aspects of maternal personality that are less functional in the role of motherhood are likely to exert an adverse influence on the emotional development of children.

Previous cross-sectional studies report that maternal neuroticism is associated with lower mother-reported parenting competence and satisfaction and offspring behavioural problems (Nigg & Hinshaw, 1998). However, there are many other aspects of personality that are not so intimately linked to the depression/anxiety/neuroticism cluster and have not been investigated in relation to offspring mental health problems. Of particular interest are personality traits associated with less affectionate parenting (Leerkes *et al.* 2015), such as suspicion (Sanz *et al.* 2010) and anger (Meier & Robinson, 2004; Ode *et al.* 2008), as well as impulsivity/sensation seeking, which are associated with inconsistent and over-reactive parenting (Chen & Johnston, 2007).

Although there is little research into the impact of these dysfunctional personality traits in population studies of mothers on child outcomes, a body of research has examined the impact of maternal personality disorders on offspring. Personality disorders represent the extreme end of dysfunctional personality traits and are associated with severe problems with interpersonal functioning. It is important to investigate the role of the underlying dysfunctional personality traits in parents, both because such traits may amplify the adverse effects of parental mental illness on a child but also because they may influence the child irrespective of parental mental health. The most heavily studied category of personality disorder in relation to parenting is Borderline Personality Disorder (BPD), a condition characterised by a pervasive pattern of instability in interpersonal relationships and self-image as well as marked impulsivity, novelty seeking, and suspicion of others. Compared with healthy mothers, previous studies have reported that mothers with BPD are less sensitive (Crandell *et al.* 2003; Newman *et al.* 2007), more intrusive (Crandell *et al.* 2003; Hobson *et al.* 2005), more hostile (Herr *et al.* 2008), and more overprotective (Feldman *et al.* 1995; Elliot *et al.* 2014) in their interactions with their offspring. Moreover, the children of mothers with BPD are more likely to display a disrupted attachment style (Abela *et al.* 2005; Hobson *et al.* 2005; Herr *et al.* 2008; Hobson *et al.* 2009; Macfie & Swan, 2009), and social withdrawal and emotion dysregulation (Crandell *et al.* 2003; White *et al.* 2011). By middle childhood, offspring of BPD mothers appear to have socio-emotional deficits (Barnow *et al*. 2006; Schacht *et al.* 2013; Elliot *et al.* 2014) as well as display a range of cognitive biases (Abela *et al.* 2005). All of these characteristics are early precursors of later common mental disorders. Two small clinical cross-sectional studies report associations between BPD in mothers and elevated risk of adolescent depressive disorders (Abela *et al.* 2005) and suicidal ideation (Barnow *et al*. 2006).

## Limitations of previous work

The majority of studies reporting adverse impacts of maternal personality traits on offspring outcomes have been limited to small clinical samples and cross-sectional designs. Clinical samples may be subject to selection bias and represent a particularly poorly functioning subset of women in need of help from clinical services. This inability to function, rather than personality dysfunction *per se*, may explain associated poorer child and parenting outcomes. Cross-sectional designs cannot provide information regarding temporal associations. Furthermore, these studies have not had adequate sample sizes or included data to separate the effects of dysfunctional personality traits from the confounding effects of surrounding adversities. Mothers with dysfunctional personality traits and disorders are more likely to parent in the context of significant additional risk factors (Barnow *et al*. 2006; Crittenden & Newman, 2010; White *et al.* 2011), and the indirect effects of those circumstances could lead to poorer child outcomes. Finally, genetic vulnerability may account for both maternal dysfunctional personality and child mental health, but prior studies have not explored this possibility. Given these methodological limitations, relations between dysfunctional maternal personality traits and later offspring outcomes at the level of the general populations remain unclear.

Using data from a large population study, we set out to investigate the relation between dysfunctional maternal personality traits measured at age 9 years and key mental health outcomes (depression, anxiety and self-harm) of their children at age 18 years. The study also aimed to investigate whether, if present, the associations were independent of maternal depression and other key surrounding adversities. The social and health burden associated with personality disorder is closely linked to the severity of underlying disturbance, as indexed by the number of underlying dysfunctional traits (Yang *et al.* 2010; Tyrer *et al.* 2015; Moran *et al.* 2016). With this in mind, we tested whether the risks of offspring depression, anxiety and self-harm were associated with the number of maternal dysfunctional personality traits. To assess the specificity of any maternal effects, we investigated the role of equivalent paternal personality traits. If genetic or environmental confounding explains associations with maternal factors, comparable associations would be expected for equivalent paternal factors. This is because father and mothers provide equal contribution to child DNA and usually share the home environment. In contrast, maternal-specific associations would provide evidence for maternal dominant environmental pathways. One potentially maternal dominate pathway is parenting given that mothers are usually the primary care-giver especially in the early years.

## Hypotheses

We set out to test the following primary hypothesis:

(1) At the level of the general population, dysfunctional maternal personality traits at child age 9 years will be associated with an increased risk of offspring mental health problems at age 18 years.
  We also set out to test the following secondary hypotheses:
(2) That greater risk will be associated with the presence of greater numbers of dysfunctional maternal personality traits.
(3) That any associations will be independent of maternal depression.

## Method

The sample comprised participants from the Avon Longitudinal Study of Parents and Children (ALSPAC), an ongoing population-based study. The study website contains details of all data available through a fully searchable data dictionary (http://www.bris.ac.uk/alspac/researchers/data-access/data-dictionary). Ethical approval for the study was obtained from the ALSPAC Ethics and Law Committee and the Local Research Ethics Committees. In total, 15 247 pregnant mothers residing in the former Avon Health Authority in the south-west of England with expected dates of delivery between 1 April 1991 and 31 December 1992 were recruited to the study. These pregnancies resulted in 14 775 live births, of which 14 701 were alive at 1 year of age. (For further details on the cohort profile, representativeness, and phases of recruitment, see Boyd *et al.* 2012; Fraser *et al.* 2012).

Here, we used data from ALSPAC mothers and offspring where mothers completed a personality assessment when the child was age 9, of these mothers 3629 offspring participants also completed the CIS-R at 18 years. Complete data for the exposure, outcome, and all covariates were available for 2793 mothers and children and 1857 fathers and children. However, using the substantial information on missing data and repeated measures we were able to impute missing data up to the sample of mothers and children with complete maternal personality data at age 9 and at least one previous measure of offspring self-harm and depressed mood (*n* = 8035).

### Parental personality at age 9

Dysfunctional personality traits in mothers and fathers were assessed using the Karolinska Scales of Personality (KSP) inventory (Gustavsson, 1997). The KSP is a self-report questionnaire measuring 15 personality traits relevant to psychological functioning and vulnerability to psychiatric disorders. It was developed to measure aspects of personality related to vulnerability for psychopathology rather than providing comprehensive coverage of all personality dimensions and has been widely used in psychopathology research. The 15 traits are measured in sub-scales for somatic anxiety, psychic anxiety, muscular tension, psychasthenia, inhibition of aggression, irritability, guilt, socialization, social desirability, monotony avoidance, impulsivity, verbal aggression, indirect aggression, suspicion and detachment. The majority of these sub-scales relate to neuroticism, a trait which itself is strongly related to state depression/anxiety (Luciano *et al.* 2012). Trait and state depression/anxiety and neuroticism are widely reported to be associated with offspring mental health. Therefore, we selected five traits *a priori* [Monotony Avoidance (novelty seeking), Impulsivity, Verbal Anger, Suspicion and Detachment] reflective of relational and affect dysregulation, and which are theoretically distinct from the neuroticism domain. Correlations between these personality traits and depressed mood are given in etable1 and as demonstrated by the relatively low correlations (*r* < 0.5), the traits are related but distinct from depressed mood. With the exception of impulsivity, maternal and paternal personality traits showed small positive correlations (ranging from r correlations of 0.1–0.2).

### Outcome measures at age 18

Children completed a self-administered computerized version of the Clinical Interview Schedule – Revised (CIS-R; Lewis, 1994). This interview assesses symptoms across multiple domains, and computer algorithms are used to identify current psychiatric disorders according to ICD-10 diagnostic criteria. This computerized version demonstrates good agreement with interviewer assessment (Lewis, 1994). The following outcomes were investigated.

#### Depressive disorder

A binary variable (depressed, not depressed); cases were those with a primary diagnosis of mild, moderate, or severe depression.

#### Anxiety disorders

A binary variable (presence, absence) of any of the following five anxiety disorders: generalized anxiety disorder, social phobia, specific (isolated) phobia, panic disorder, or agoraphobia according to ICD-10 criteria.

#### Self-harm

Assessed using the CIS-R, participants were classified as having a lifetime history of self-harm if they responded positively to the question ‘have you ever hurt yourself on purpose in any way (e.g. by taking an overdose of pills or by cutting yourself)?’

#### Potential confounding variables

We adjusted on *a priori* grounds for the following socio-demographic and family factors: maternal education (highest level achieved), maternal age at child birth (years), maternal binge drinking in offspring's early childhood (frequency mother drinks more than 4 units of alcohol), maternal depression during the postnatal period taken as the average score on the Edinburgh Postnatal Depression Scale (EPDS) measured at 2 months and 8 months postpartum as used in previous studies (see Stein *et al.* 2014) , maternal smoking (mother ever smoked), financial difficulties, family income, maternal reports of intimate partner violence and child gender.

### Statistical analysis

First, we conducted a series of separate logistic regression analyses to test associations between each maternal personality trait (standardised continuous scores) and risk of offspring self-harm, depression, and anxiety. These models were repeated, mutually adjusting for other personality traits to investigate whether any particular trait-outcome association was independent of the effects of other personality traits. These models were repeated in the same way for paternal personality traits.

To investigate the cumulative impact of combined maternal personality traits, we grouped women's scores on each personality trait into quartiles. We then identified women having a score in the top quartile as being high on that trait and derived a count of the number of top quartile personality traits. This ordinal variable was then regressed on each outcome in further logistic regression models. The risk of outcomes at each level of this variable and the linear association across levels were explored. Finally, we adjusted all associations for potential confounding variables.

**Fig. 1.**
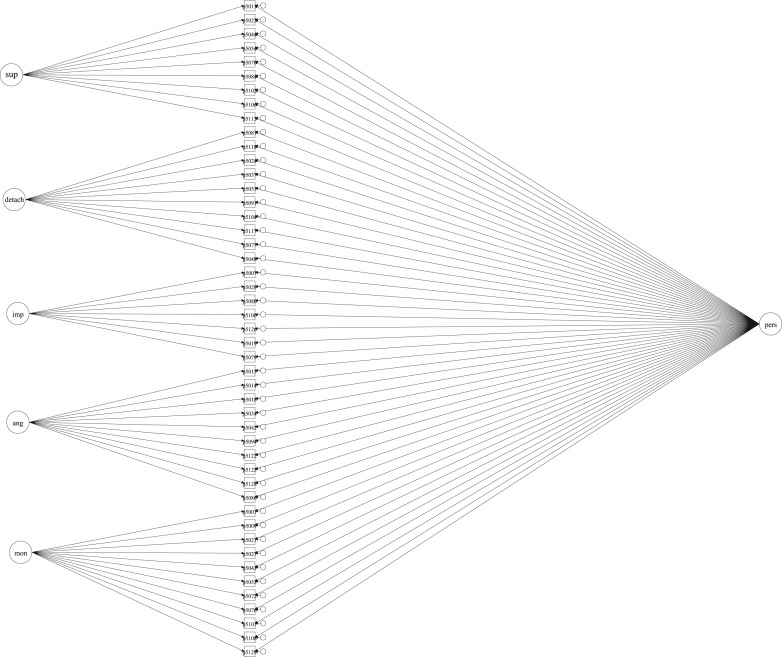
Representation of bi-factor latent trait for personality dysfunction (general factor on the right) and specific traits (on the left).

To extend these analyses and further understand the role of having high levels across all dysfunctional personality traits, rather than the effects of each trait in isolation, we derived a latent factor representing the shared variance in all personality traits. Individuals who are high on this latent variable would show high scores across *all* traits. We initially derived latent factors for each of the five maternal personality constructs using confirmatory factor analysis. To model the variance shared amongst these factors, a bi-factor, latent variable based on shared variance among these five factors was derived by cross loading all items onto their specific factor as well as a general factor (using confirmatory factor analysis), see Fig. 1. Model fit for this variable was good RMSEA < 0.01 and CFI > 0.8. This model is shown in Fig. 1. We then explored the association between this general latent factor for personality dysfunction and observed binary variables as above for depression, anxiety, and self-harm at 18, using a weighted least squares (WSLMV) estimator due to categorical outcomes. However, to aid interpretation of the latent approach alongside the regression models, we also extracted the factor score generated from the latent model and regressed this onto the binary outcomes using logistic regression models.

Characteristics of the complete case sample compared with partial responders and the rest of the ALSPAC sample have been explored in detail elsewhere and there is evidence that missing data are predictable from partial observed data. We therefore examined the impact of missing data on our results by repeating analyses using 60 datasets with multiply imputed missing values by chained equations (Royston, 2009; see online Supplement for full details). We imputed up to a sample with complete *maternal* personality measures and at least one offspring mood and self-harm measure at any time point (*n* = 8035). All analyses were undertaken using Stata v15 and MPlus (for the latent variable analysis).

## Results

Characteristics of mothers according to number of dysfunctional personality traits are given in Table 1. There were clear dose-response associations between the number of high dysfunctional maternal personality traits and high-risk maternal characteristics, with particularly noticeable differences in mothers with three or more high personality traits.

**Table 1.** Frequencies and characteristics of mothers with increasing numbers of high personality dysfunction traits

	Low/moderate on all personality traits *N* = 3290 (42%)	High on 1 *N* = 2024 (26%)	High on 2 *N* = 1301 (17%)	High on 3+ *N* = 1248 (16%)	*p* value for differences across groups, using χ^2^
Low maternal education (only GCSES)	*N* = 1620 (54%)	*N* = 977 (55%)	*N* = 646 (57%)	*N* = 640 (64%)	*p* < 0.001
Low family income (based on median split of absolute income)	*N* = 1329 (49%)	*N* = 774 (48%)	*N* = 528 (52%)	*N* = 545 (62%)	*p* < 0.001
Financial problems	*N* = 453 (14%)	*N* = 363 (19%)	*N* = 228 (19%)	*N* = 267 (25%)	*p* < 0.001
Young mother at birth of child (<20 years)	*N* = 138 (4%)	*N* = 115 (6%)	*N* = 86 (7%)	*N* = 114 (10%)	*p* < 0.001
Daily drinking postnatally (+4 units)	*N* = 77 (3%)	*N* = 56 (3%)	*N* = 27 (3%)	*N* = 56 (6%)	*p* < 0.001
Postnatal depression (>12 EPDS score)	*N* = 126 (4%)	*N* = 155 (8%)	*N* = 149 (13%)	*N* = 197 (19%)	*p* < 0.001
Maternal smoking (ever smoked)	*N* = 1554 (51%)	*N* = 947 (52%)	*N* = 626 (55%)	*N* = 631 (62%)	*p* < 0.001
Maternal suicide attempt (mother reported)	*N* = 21 (0.7%)	*N* = 23 (1 %)	*N* = 27 (2%)	*N* = 52 (5%)	*p* < 0.001
Maternal self-harm (child reported)	*N* = 16 (0.8%)	*N* = 11 (1%)	*N* = 2 (0.3%)	*N* = 25 (4%)	*p* < 0.001
Never married	*N* = 278 (9%)	*N* = 213 (12%)	*N* = 143 (12%)	*N* = 190 (19%)	*p* < 0.001
No partner during pregnancy	*N* = 19 (1%)	*N* = 10 (1%)	*N* = 8 (1%)	*N* = 18 (2%)	*p* = 0.009
Physically abused by partner (mother reported)	*N* = 93 (3%)	*N* = 77 (4%)	*N* = 62 (5%)	*N* = 103 (10%)	*p* < 0.001
Emotionally abused by partner (mother reported)	*N* = 157 (5%)	*N* = 118 (6%)	*N* = 101 (9%)	*N* = 150 (15%)	*p* < 0.001
Mum sexually abused as child (mother reported)	*N* = 79 (3%)	*N* = 78 (4%)	*N* = 66 (6%)	*N* = 79 (7%)	*p* < 0.001
Mum physically abused as child (mother reported)	*N* = 83 (3%)	*N* = 84 (5%)	*N* = 80 (7%)	*N* = 111 (11%)	*p* < 0.001

### Associations between individual maternal personality traits and offspring mental health

#### Main effects

As shown in Table 2, higher scores across most dysfunctional maternal personality traits were associated with increased risk of offspring mental health problems. Mutually adjusted models suggested that most associations were not independent. However, high levels of maternal suspicion had an independent effect across outcomes and maternal impulsivity had an independent effect on offspring depression.

**Table 2.** Logistic regression to test associations between maternal personality traits (standardised continuous scores) and risk of offspring self-harm, depression, and anxiety at 18, firstly in separate models and then mutually adjusted for other personality traits

	Odds ratio (95% CI) for child self-harm at 18		Odds ratio (95% CI) for child depression at 18		Odds ratio (95% CI) for child anxiety at 18	
	Unadjusted	Mutually adjusted	Unadjusted	Mutually adjusted	Unadjusted	Mutually adjusted
Monotony avoidance	1.14 (1.05–1.24) *p* = 0.003	1.06 (0.95–1.17) *p* = 0.289	1.21 (1.07–1.35) *p* = 0.002	1.12 (0.99–1.28) *p* = 0.082	1.04 (0.89–1.22) *p* = 0.589	1.00 (0.84–1.19) *p* = 0.972
Suspicion	1.21 (1.11–1.32) *p* = 0.000	1.17 (1.06–1.3) *p* = 0.002	1.23 (1.09–1.38) *p* = 0.001	1.15 (0.99–1.35) *p* = 0.072	1.29 (1.11–1.5) *p* = 0.001	1.19 (0.98–1.46) *p* = 0.081
Impulsivity	1.07 (0.98–1.17) *p* = 0.112	0.98 (0.89–1.09) *p* = 0.749	1.25 (1.11–1.4) *p* = 0.000	1.17 (1.03–1.33) *p* = 0.014	1.1 (0.94–1.27) *p* = 0.227	1.07 (0.91–1.26) *p* = 0.424
Anger	1.14 (1.04–1.24) *p* = 0.005	1.08 (0.97–1.19) *p* = 0.144	1.15 (1.02–1.4) *p* = 0.021	1.04 (0.91–1.19) *p* = 0.558	1.03 (0.88–1.2) *p* = 0.747	0.97 (0.82–1.16) *p* = 0.752
Detachment	1.09 (1.00–1.19) *p* = 0.046	1.08 (0.99–1.18) *p* = 0.092	1.16 (1.03–1.3) *p* = 0.013	1.05 (0.91–1.2) *p* = 0.508	1.2 (1.04–1.39) *p* = 0.014	1.12 (0.94–1.33) *p* = 0.219

As shown in Table 3, there was no evidence for an association between any dysfunctional personality traits in fathers and offspring mental health. We did not explore paternal variables in any further analyses.

**Table 3.** Logistic regressions to test associations between paternal personality traits (standardized continuous scores, so the OR represent increased odds for each 1 s.d. increase in the score) and risk of offspring self-harm, depression, and anxiety at 18.

Paternal traits	Odds ratio (95% CI) for child self-harm at 18	Odds ratio (95% CI) for child depression at 18	Odds ratio (95% CI) for child anxiety at 18
Monotony avoidance	1.11 (0.98–1.26) *p* = 0.089	0.85 (0.70–1.02) *p* = 0.084	1.12 (0.90–1.39) *p* = 0.325
Suspicion	1.07 (0.94–1.22) *p* = 0.317	0.88 (0.72–1.07) *p* = 0.186	1.08 (0.86–1.35) *p* = 0.511
Impulsivity	1.10 (0.97–1.25) *p* = 0.130	0.87 (0.72–1.05) *p* = 0.150	1.13 (0.91–1.40) *p* = 0.275
Anger	1.08 (0.95–1.23) *p* = 0.215	0.83 (0.69–1.01) *p* = 0.056	1.06 (0.84–1.33) *p* = 0.616
Detachment	0.98 (0.86–1.12) *p* = 0.756	0.96 (0.79–1.15) *p* = 0.643	0.91 (0.72–1.15) *p* = 0.452

### The impact of mothers having multiple dysfunctional personality traits on offspring mental health

#### Accumulation of high traits

**Fig. 2.**
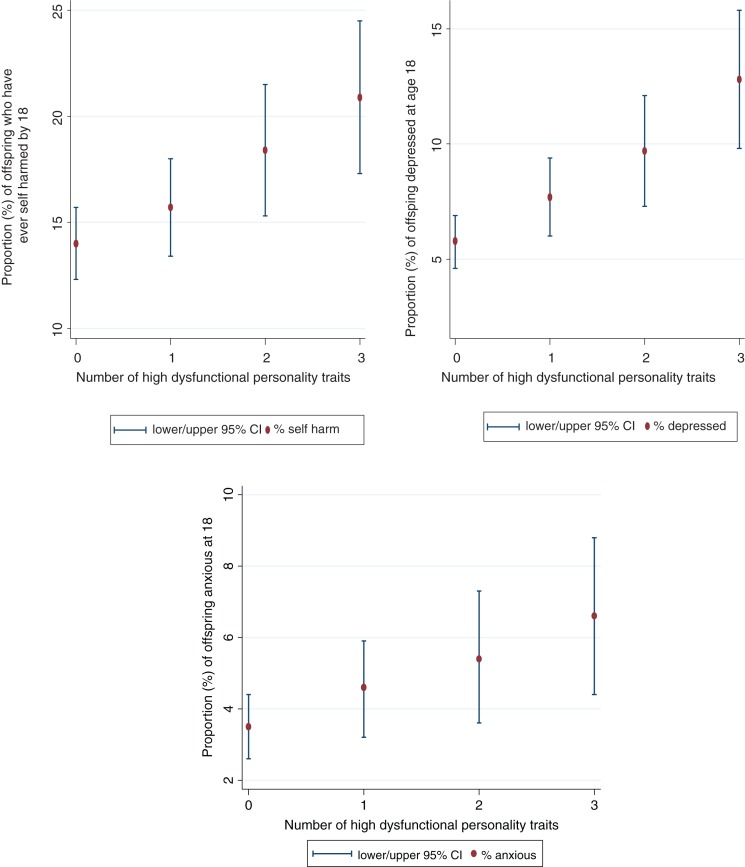
Percentage of offspring with self-harm, depression and anxiety disorders at 18 years of age, according to number of maternal personality traits.

As shown in Table 4, dose-response associations emerged between the number of high dysfunctional maternal personality traits and offspring risk of self-harm, depression, and anxiety. There was evidence that associations with self-harm and anxiety weakened with confidence intervals (CIs) including the null following adjustments, indicating that these associations may be confounded by surrounding adversities. With the additional power (and reduction of bias) in the post-imputation sample, however, we found evidence for an association with self-harm. Clear associations were observed for offspring depression even after including adjustment variables and in both complete case and imputed data (Fig. 2).

**Table 4.** Logistic regressions to test associations between the number of dysfunctional maternal personality traits and offspring self-harm, depression, and anxiety at 18. Complete cases (n = 2793 in all models) and post imputation for missing data (n = 8035)

	Odds ratio (95% CI) for child self-harm by 18			Odds ratio (95% CI) for child depression by 18			Odds ratio (95% CI) child anxiety at 18		
	Unadjusted	Adjusted^a^	Adjusted model	Unadjusted	Adjusted^a^	Adjusted model	Unadjusted	Adjusted^a^	Adjusted model
Low/moderate on all personality dysfunction	REF	REF	REF	REF	REF	REF	REF	REF	REF
High on 1	1.14 (0.88–1.49)	1.11 (0.85–1.46)	1.10 (0.92–1.32)	1.42 (0.98–2.07)	1.42 (0.97–2.08)	1.28 (0.99–1.65)	1.44 (0.9–2.31)	1.35 (0.83–2.18)	1.15 (0.81–1.64)
High on 2	1.39 (1.03–1.86)	1.28 (0.94–1.73)	1.26 (1.14–1.58)	1.82 (1.22–2.72)	1.77 (1.17–2.68)	1.57 (1.17–2.11)	1.69 (1.01–2.83)	1.43 (0.84–2.44)	1.34 (0.92–2.07)
High on 3+	1.44 (1.04–1.99)	1.26 (0.89–1.8)	1.34 (1.26–1.75)	2.33 (1.53–3.53)	2.27 (1.45–3.54)	1.94 (1.39–2.7)	1.85 (1.06–2.23)	1.38 (0.76–2.5)	1.38 (0.89–2.16)
Linear trend	*p* = 0.007	*p* = 0.088	*p* = 0.014	*p* < 0.001	*p* < 0.001	*p* < 0.001	*p* = 0.012	*p* = 0.194	*p* = 0.090

a Maternal education (highest level achieved), maternal age (years), maternal binge drinking in offspring's early childhood (frequency mother drinks more than 4 units of alcohol), maternal depression (EPDS), maternal smoking (mother ever smoked), child gender, family income, financial problems and mothers report of partner violence during the index child's childhood.

#### Latent variable approach

We explored the association between the shared variance latent variable for dysfunctional personality traits (Fig. 1) and self-harm, depression, and anxiety using regression in a structural equation model using Mplus. There was strong statistical evidence that this latent variable was associated with offspring mental health outcomes, standardised path coefficient (can be interpreted as correlations) =0·161 *p* < 0·001 for depression; 0·159 *p* < 0·001 for self-harm; and 0·141 *p* = 0·001 for anxiety disorders. We also investigated the association between the saved factor score and outcomes in a logistic regression model to aid comparability with the main analysis described above, finding that a 1 s.d. increase in the factor score for personality dysfunction was associated with depression (odds ratio (OR) 1.4 (1.2–1.4 *p* < 0.001), self-harm (OR 1.3 (1.2–1.4, *p* < 0.001) and anxiety (OR 1.5 (1.3–1.7, *p* < 0.001). This analysis further supports the interpretation that the combined variance associated with these dysfunctional personality traits is most important for offspring mental health.

## Discussion

In this large-population cohort, high levels of dysfunctional maternal personality traits in middle childhood were associated with increased risk of serious mental health problems in offspring on the cusp of adulthood. The associations were strongest for offspring depression, but similar patterns were seen for self-harm and anxiety disorders. There was some evidence for independent associations between impulsivity and suspicion and offspring depression. Overall, however, the risk was best explained by the combination of multiple dysfunctional maternal personality traits. Indeed, there was a clear dose response association with increased numbers of high dysfunctional traits in mothers. The latent variable analysis provided further evidence of the importance of the combined variance between all five maternal traits. Associations were independent of maternal depression and other high-risk maternal characteristics and did not hold for fathers.

### Strengths and limitations

To our knowledge this is the first prospective large-scale longitudinal study of the long-term impact of dysfunctional maternal personality traits on risk of offspring mental disorders. Strengths of the study include the large population-based sample, longitudinal design, inclusion of a wide range of covariates, use of regression modelling to adjust for confounding, and latent variable analysis to examine the impact of shared variance between personality traits and offspring outcomes. There are also a number of limitations. First, genetic data were not included in the analysis. It is possible that shared genetic risk of psychopathology, which may be expressed as different phenotypes in mothers and offspring, explains the observed associations. However, the fact that there was no evidence for any association with paternal personality traits makes a genetic pathway less likely, given that offspring receive half of their genome from their fathers. In addition, although we accounted for several confounding factors, residual confounding is always likely in observational studies and particularly for exposures such as personality, which are associated with a complex array of environmental and genetic factors. In addition, there was no measure of maternal depression taken at the same time as the personality measures, however, we did adjust for maternal depression measured early in the child's life. We were also not able to look at parenting measures. Although ALSPAC recorded parenting, these data were collected many years prior to the assessment of personality, and thus did not allow us to investigate the mediating effect of parenting. It would be important to investigate the mediating role of parenting in future studies of personality and offspring outcomes.

Another limitation is the potential role of bias due to high attrition in ALSPAC. However, given that there is substantial information on the characteristics of mothers and offspring who drop out, the nature of this bias can be explored by using this existing information to impute missing values. Results were similar using imputed data suggesting that the effects of this potential bias were not substantial.

### Potential explanations and mechanisms

Observational studies alone cannot provide evidence that maternal personality traits cause offspring mental health problems. However, some informed speculation of mechanisms is possible. Mothers with dysfunctional personality traits may live in adverse circumstances and also engage in a range of unhealthy behaviours. Thus, it may be the case that the observed associations reflect exposure to a nexus of adversity rather than the influence of dysfunctional personality traits *per se*. That said, we adjusted for a wide range of variables in regression models, and the associations remained relatively unchanged, suggesting that surrounding adversities did not account for maternal personality-offspring risk associations.

There are three main areas in which dysfunctional maternal personality traits may manifest in behaviour causing difficulties for the child's emotional development.

First, suspiciousness and detachment on the part of the mother may result in unavailability and disengagement from the child. During interactions with their children, mothers with BPD have been reported to smile less, play fewer games (White *et al.* 2011) and to be less emotionally available for their children (Hobson *et al.* 2005; Delavenne *et al.* 2008). Lack of engagement in turn is associated with poor attachment and emotional development in children. Support with and validation of negative emotions appears to be particularly relevant in the development of self-harm (Nock, 2009), and this connection may explain why self-harm outcomes were associated with suspiciousness in mothers.

Second, inconsistent or chaotic maternal parenting (i.e. behaviours that oscillate between high stimulation and disengagement) may be a consequence of underlying impulsivity (Chen & Johnston, 2007). Such impulsivity may lead to the child feeling insecure and uncontained. Over time this may manifest in depression and anxiety – an assertion supported by animal models of anhedonic behaviour (Baram *et al.* 2012).

Finally, harsh punishment and hostile parenting may be related to angry, impulsive, and suspicious traits in mothers. Harsh punishment is associated with elevated stress and poor emotional regulation in offspring, which may, over time, manifest as self-harm, depression, and anxiety (Hallquist *et al.* 2015).

The finding of no associations between paternal personality traits and offspring mental health may be surprising given that (possibly due to assortative mating), there was a small positive correlation between maternal and paternal personality. The lack of association may be explained by personality traits having a different ‘meaning’ or manifestation in mothers and fathers and thus a different impact on parenting. Our findings could suggest that the personality traits found to be less optimal in the mothering role, should not necessarily be considered as risk factors in fathers.

### Implications

The current findings demonstrate the potential importance of supporting mothers with high levels of dysfunctional personality traits. Although we did not explore parenting in this study, we hypothesize that dysfunctional maternal personality traits are at high risk of leading to disengaged, inconsistent, and hostile parenting behaviour. The presence of such personality traits could be used to flag specific maternal support needs. The acceptability of routinely identifying this population of mothers requires empirical testing – the potential risks of stigma need to be weighed against the gains (resulting from early effective help). Moreover, an effective intervention is likely to require multiple components tackling not only maternal dysfunctional personality traits (for example by using elements of established treatments such as Dialectical Behavioural Therapy or Mentalization-Based Therapy) but also surrounding environmental adversities, as well as the specific parenting challenges. Nevertheless, our findings shed important light on a hitherto neglected population of mothers and their offspring, whose needs require greater scientific understanding and wider societal acknowledgement.

## Supplementary material

For supplementary material accompanying this paper visit http://dx.doi.org/10.1017/S0033291717001246. click here to view supplementary material
