# Supplementary material for: Impact of dysfunctional maternal personality traits on risk of offspring depression, anxiety and self-harm at age 18 years: a population-based longitudinal study
Source: Psychol Med. 2017 Jun 6;48(1):50–60. doi: 10.1017/S0033291717001246 (PMC5729843; doi:10.1017/S0033291717001246)
Supplement: Supplementary file 1 [file S0033291717001246sup001.docx]

**SUPPLEMENT**

**Further details on items in the KSP**

Anger items were taken from the verbal aggression scale of the KSP. Items on this subscale relate to outward displays of anger, such as shouting. For example, ‘when someone is pushing themselves forward in a queue, I usually tell them off’. There are also items relating to more subtle displays of verbal aggression. The item ‘I sometimes spread gossip about people I don’t like’ reflects this. The Detachment subscale captures the lack of a person’s willingness and comfort in opening up and becoming close to others. For example, ‘I feel best when I keep people at a distance’. The suspicion subscale, relates to being distrusting of other people’s motives. For example, ‘I commonly wonder what hidden reason another person might have for doing something nice for me’. Impulsivity relates to a person’s tendency to act quickly, without thinking or forming a plan. An example of an item from this scale is, ‘I have a tendency to act on the spur of the moment without really thinking ahead’. High monotony avoidance indicates an increased need for excitement and change. For example, ‘I almost always have a desire for more action’.

**Table e1. Correlation matrix of maternal personality traits and depressed mood to indicate overlap.**

|  | **Monotony Avoidance** | **Suspicion** | **Impulsivity** | **Anger** | **Detachment** | **Depressed mood** |
| --- | --- | --- | --- | --- | --- | --- |
| **Monotony Avoidance** | -- |  |  |  |  |  |
| **Suspicion** | 0.32 | -- |  |  |  |  |
| **Impulsivity** | 0.43 | 0.31 | -- |  |  |  |
| **Anger** | 0.36 | 0.32 | 0.36 | -- |  |  |
| **Detachment** | 0.11 | 0.5 | 0.15 | 0.13 | -- |  |
| **Depressed mood** | 0.26 | 0.47 | 0.24 | 0.22 | 0.27 | -- |

Correlations amongst individual traits are given in Table e1. The individual traits were related to each other but at a level which highlighted independence because less than 25% of the variance is shared (i.e., correlation <0·5). All traits are correlated with depressed mood but again should independence.

**Missing data information.**

Multiply imputed missing values by chained equations is recommended for missing data (Sterne et al., 2009) and assumes that data are missing at random (MAR) taking into account the variables in the imputation model. To ensure plausibility of the MAR assumption, we included a number of auxiliary variables predictive of incomplete variables and/or missingness in the imputation model, including additional socio-demographic variables, mental health assessments, and mood scales, and self-reports of self-harm at additional time-points (full list available on request).
